# Adverse Events and Complications in Continuous Intra-arterial Nimodipine Infusion Therapy After Aneurysmal Subarachnoid Hemorrhage

**DOI:** 10.3389/fneur.2021.812898

**Published:** 2022-02-18

**Authors:** Thomas Kapapa, Ralph König, Benjamin Mayer, Michael Braun, Bernd Schmitz, Silwia Müller, Julia Schick, Christian Rainer Wirtz, Andrej Pala

**Affiliations:** ^1^Department of Neurosurgery, University Hospital Ulm, Ulm, Germany; ^2^Department of Neurosurgery, University of Ulm, Bezirkskrankenhaus Günzburg, Günzburg, Germany; ^3^Institute of Epidemiology and Medical Biometry, University of Ulm, Ulm, Germany; ^4^Section Neuroradiology, University Hospital Ulm, Günzburg, Germany; ^5^Section Interdisciplinary Operative Intensive Care Medicine, University Hospital Ulm, Ulm, Germany

**Keywords:** delayed cerebral ischemia, cerebral vasospasm, risk factors, endovascular therapy, outcome

## Abstract

**Objective:**

To determine the frequency and severity of complications associated with the continuous intra-arterial infusion of nimodipine (CIANI) as a new treatment of delayed cerebral ischemia (DCI) after aneurysmal subarachnoid hemorrhage (SAH).

**Methods:**

Patients from two centers (*n* = 718) treated for SAH between 2008 and 2016 were included. Demographic and SAH-related parameters were evaluated, and also the frequency of adverse events (AEs) and complications including their severity (mild, moderate, and severe). Clinical outcome was analyzed using Glasgow Outcome Scale (GOS). The unfavorable outcome was defined as GOS 1 to 3, and favorable outcome as GOS 4 to 5. The Short-Form 36 (SF-36) health-related quality-of-life (QoL) questionnaire served as a QoL measurement.

**Results:**

Of 718 patients, 65 (9%) were treated by CIANI and had a higher clinical or imaging grade of bleeding severity. Clinical deterioration while on treatment happened more often in patients who were treated with CIANI than in others. In patients with CIANI, 67% had AEs and/or complications during the treatment. Nimodipine-associated hypotension was seen in 8% (mild). Catheter-associated thrombus occurred in 9% (moderate). New intracerebral hemorrhage was found in 14% (moderate). A total of 6% treated by CIANI died during the treatment period (severe). More than one-third (39%) of patients of CIANI reached at least moderate disability, and 23% showed good recovery. Patients who received CIANI showed reduced QoL, but differences in mental and general health, and also pain were minimal.

**Conclusion:**

Patients who received CIANI had higher rates of AEs and complications. However, this does not exclude the possibility that the use of CIANI might be helpful in patients with severe and therapy-refractory CV and DCI. Controlled and randomized studies would be helpful to clarify this question but they are methodologically and ethically challenging.

## Introduction

In the treatment of aneurysmal subarachnoid hemorrhage (SAH) a new neurological deficit and impaired consciousness, lasting more than 1 h, without direct connection to hydrocephalus, rebleeding or infectious situations, or the appearance of new ischemia or infarcts displayed in adequate imaging studies is defined as delayed cerebral ischemia (DCI) (1). Up to 30% of patients with SAH develop DCI (2). In-hospital mortality rates of up to 13% are reported due to DCI (3). Many survivors remain dependent on daily life activities. However, effective protective measures and adequate treatment for DCI are still the subject of research (4, 5).

Nimodipine represents the only proven standard treatment regimen to prevent and treat cerebral vasospasm (CV) and DCI (6, 7). New salvage therapeutic strategies such as intravenous, intra-arterial, or continuous intra-arterial infusion of calcium channel blockers have been introduced recently in the case of therapy refractory CV and DCI (8–11). Continuous intra-arterial nimodipine infusion (CIANI) might be beneficial for cerebral perfusion pressure and prolong the vasodilatory effect of calcium channel blockers (12). Furthermore, CIANI has been proposed to improve outcomes in patients with severe therapy refractory CV and DCI (8).

Nevertheless, the necessity of permanent anticoagulation and inhibition of platelets aggregation as well as the influence of nimodipine and vasopressors might result in many adverse events (AEs) and complications (13, 14). This comparison of a life-threatening, complicated course of DCI vs. an effective but potentially complication-prone therapy has led to diverse discussions about the benefit of this new method. Nevertheless, the arising question is whether treatment-related complication risk using the CIANI is comparable with the mortality and morbidity of the natural course of severe DCI (14).

In order to estimate the rate of AEs and complications of CIANI, we performed an assessment of the frequency and severity in patients treated for SAH between 2008 and 2016. The focus of this work is on a description of the frequency of AEs and complications in the use of CIANI. Quality-of-life data in patients with SAH are generally spare and considering the invasive salvage therapy, a detailed analysis of independence in daily life, cognitive, emotional, and social dysfunctions has not been performed so far. Hence, the Glasgow outcome scale (GOS) and also the health-related QoL in patients who received CIANI were evaluated.

## Methods

### Study Design

This retrospective, descriptive, observational study included all consecutive patients with SAH treated in two centers between 2008 and 2016. The study was approved by the Local Ethics Committee (No. 168/17) and performed according to the international Declaration of Helsinki.

### Standard Operating Procedures and Salvage Therapy of DCI

Confirmation of SAH was performed by MRI, CT, and/or lumbar punction. An aneurysmal source of bleeding was suspected in case of hemorrhage into the basal subarachnoid cisterns, the cerebral parenchyma, or subarachnoid regions close to larger arterial vessels with a typical pattern. The source of bleeding was detected by CT-angiography or digital subtraction angiography (DSA) in case of aneurysmal SAH. Acute hydrocephalus was assumed in the presence of an enlarged ventricular system, narrowed apical sulci without signs of brain edema, and signs of liquor-diapedesis. In the case of hydrocephalus, an external ventricular drainage was implanted and if an aneurysm was identified as the source of SAH it was sealed within 24 h. The decision-making for the most appropriate aneurysm treatment modality was done by a neurovascular team consisting of at least of one experienced neurovascular radiologist and at least one experienced vascular neurosurgeon. Postinterventional or postsurgical therapy took place at an intensive care unit (ICU).

**Figure 1:**
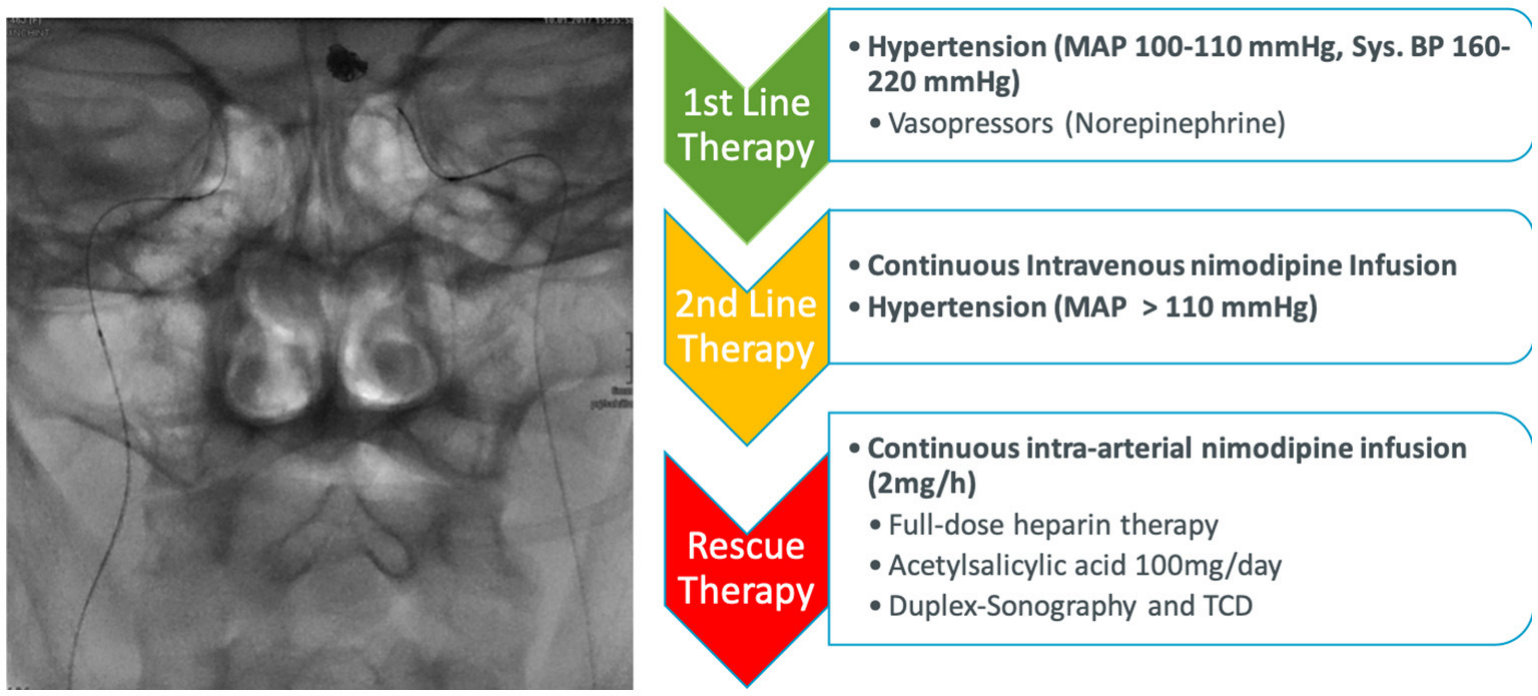
Treatment pathway of delayed cerebral ischemia and cerebral vasospasm. All patients received 60 mg nimodipine orally every 4 h after admission. In the case of progression cerebral vasospasm (CV) and delayed cerebral ischemia (DCI) the nimodipine was switched to intravenous application and strict induced hypertension was started. In the case of further therapy-refractory CV and DCI resulting in perfusion deficit and/or severe neurological impairment continuous intra-arterial nimodipine therapy was started.

All SAH patients received nimodipine 6 × 60 mg orally (60 mg every 4 h) since admission. Transcranial Doppler ultrasound (TCD) and neurological assessment represent daily measures one to three times a day. In case of clinical deterioration as a consequence of DCI or relevant increase of blood flow velocity in TCD because of the severe vasospasm, oral nimodipine dose was transformed into continuous intravenous nimodipine application (0.2 mg/ml, maximal dose 2 mg/h). Norepinephrine was standardly used to induce arterial normotension or hypertension in the case of DCI (15). In the case of CV and DCI mean arterial pressure (MAP) of 100–110 mmHg and in the case of further deterioration MAP > 110 mmHg was targeted. A MAP > 110 mm Hg was aimed during CIANI treatment. In addition, euvolemia, normal glucose blood levels (80–150 mg/dl) and also normothermia were strictly indicated (15) (Figure 1).

Daily interruption, early stop of sedation, and early weaning from mechanical ventilation as soon as possible were the overriding principle in intensive care after primary treatment of aneurysm and hydrocephalus (16, 17). In patients in the need of further sedation and ventilation, intracranial pressure (ICP) monitoring and tissue oxygen partial pressure (ptiO_2_) measurements were additionally indicated. An intraparenchymal ICP and ptiO_2_ probe was implanted.

Cerebral vasospasm was defined as increased blood flow velocity in TCD of the basal arterial vessels of more than 120 cm/s, increase of blood flow velocity of more than 30% a day, >30 cm/s/day, or vessel narrowing in CT or DSA (18). If progressive DCI despite hypertension and intravenous application of nimodipine was suspected, a CT-angiogram and CT-perfusion were performed. In the presence of new infarction or a perfusion delay, defined as a mismatch between reduced mean transit time (MTT) (>4 s) and adequate cerebral blood volume (CBV), DSA, and continuous intra-arterial instead of intravenous infusion of nimodipine *via* indwelling microcatheters in both internal carotid arteries was indicated (Figure 1) (11). Nimodipine-associated hypotension was defined as any hypotension in temporally or in dosage connection to the administration of nimodipine of more than 10 mmHg for more than 15 min.

### Patients

A total of 718 patients were considered for the analyses. They were rated clinically based on the World Federation of Neurosurgical Societies Scale (WFNS) and Hunt & Hess scale (HH) and accordingly divided into groups: I–III mild SAH and IV-V severe SAH (19, 20). The Fisher Scale classified the intensity of the initial hemorrhage, with scores between 1 (no blood) and 4 (intraventricular, intracerebral blood) (21). The localization of the bleeding source was divided into the groups “anterior circulation” (anterior cerebral artery, anterior communicating artery, posterior communicating artery, internal carotid artery, and middle cerebral artery), “posterior circulation” (posterior cerebral artery, vertebral artery, posterior inferior cerebellar artery, and basilar artery) and “SAH with negative angiography.”

### Definition of Adverse Events and Complications

Adverse events were defined as suggested by the international conference on harmonization of technical requirements for registration of pharmaceuticals for human use: “Any untoward medical *occurrence* in a patient or clinical investigation subject administered a pharmaceutical product and which does not necessarily have to have a causal relationship with this treatment. An AE can therefore be any unfavorable and unintended sign (including an abnormal laboratory finding, for example), symptom, or disease temporally associated with the use of a medicinal product, whether or not considered related to the medicinal product” (22). However, we defined any ongoing AEs (occurrence, sign or symptom) as a complication. Complications were defined as “[…] an *ongoing* unfavorable evolution or consequence of a disease, a health condition or a therapy. The disease can become worse in its severity or show a higher number of signs, symptoms or new pathological changes, become widespread throughout the body or affect other organ systems. […] A medical treatment, such as drugs or surgery may produce adverse effects or produce new health problem(s) by itself. Therefore, a complication may be iatrogenic […]” (23).

The severity of AEs and complications was divided into three levels according to the measures to be taken and the influence on the outcome. Mild AEs and complications are short-termed, require little intervention, and have no lasting impact on the outcome. Moderate AEs and complications are permanent, but their effects can be changed and alleviated through medical intervention. Their influence on the treatment result is limited. Severe AEs and complications are permanent and have a serious influence on the outcome, e.g., by at least one category of GOS.

### Clinical Outcome Analysis

Clinical outcome was measured using the Glasgow Outcome Scale (GOS) 6 months after the treatment period. A standardized, generic survey for health-related QoL was chosen to ensure reproducibility and comparability in this topic. All patients who received treatment of CIANI received the 36-Item Short Form Health Survey (SF-36) (24).

### Neuroimaging Analysis

Two independent neuroradiologists evaluated the radiological imaging and reports of all patients right before and after treatment of CIANI with regard to newly occurred cerebral infarcts during therapy. First, all the patients were screened on pre-existing, acute infarctions in advance of CIANI therapy. Possible new infarctions after therapy were recorded subsequently.

### Data Collection and Statistical Analysis

All the aforementioned data were collected from clinical files, the picture archiving, and communicating system, and stored in an electronic database (Microsoft, Excel 2010, Redmond, USA). Data were collected by three scientific members of the departments.

Data were initially analyzed by descriptive methods. Arithmetic mean, SD, and range were used to describe quantitative parameters, whereas absolute and relative frequencies were calculated for qualitative parameters. For comparisons between interesting subgroups an independent *t*-test was used in the case of normally distributed quantitative data, and the non-parametric alternative of Mann-Whitney U-test otherwise. Fisher's exact or chi-squared tests were used for the comparison of qualitative data. In order to account for potential confounding of the results from statistical hypothesis testing, multiple regression analysis was used. The explorative significance level was defined as *p* ≤ 0.05. Data were analyzed using SPSS 25 (IBM Corporation, Armonk, USA).

## Results

A total of 65 patients out of the total 718 patients underwent CIANI (9%). Demographic data are summarized in Table 1. Patients with CIANI were significantly younger (*p* = 0.004) and the majority had the source of bleeding at the anterior circulation (*p* = 0.001). Only one patient without an identified source of bleeding received CIANI therapy. Endovascular treatment was performed in the majority of cases (75%). The distributions of Fisher score, WFNS score, and HH grades in both cohorts are depicted in Table 2. Patients with Fisher score IV received CIANI therapy more frequently (*p* < 0.01). There was no significant difference in WFNS grades II–V and Fisher grade III between treatment of CIANI and non-CIANI. In patients with WFNS I, CIANI was less common.

**Table 1:** Patients' clinical characteristics.

	**Conservative**	**CIANI**	***p***
*n*	91% (653)	9% (65)	
Mean age (years)	55.6 (SD 13.5)	52.6 (SD 11.5)	0.004
Female ratio	62% (405)	63% (41)	0.8
SAH with negative angiography	27% (179)	2% (1)	<0.001
Anterior circulation	54% (350)	77% (50)	0.001
Posterior circulation	16% (103)	21% (14)	0.3
Not defined	3% (21)		
Endovascular aneurysm intervention	82% (389)	75% (49)	0.02
Surgical clipping	18% (85)	23% (15)	0.04
Cerebral vasospasm and DCI	34% (217)	100% (65)	

**Table 2:** Initial grading scores of patients with subarachnoid hemorrhage.

**Hunt and Hess grade**	**Conservative (*n* = 646)**	**CIANI (*n* = 65)**	***p***
I	19% (124)	8% (5)	0.03
II	38% (243)	26% (17)	0.09
III	19% (120)	29% (19)	0.05
IV	15% (96)	28% (18)	0.01
V	9% (63)	9% (6)	0.8
**WFNS**	**Conservative** **(*****n*** **=** **648)**	**CIANI** **(*****n*** **=** **65)**	***p***
I	49% (314)	28% (18)	<0.01
II	13% (85)	12% (8)	0.85
III	4% (27)	9% (6)	0.12
IV	6% (37)	11% (7)	0.18
V	28% (185)	40% (26)	0.07
**Fisher score**	**Conservative** **(*****n*** **=** **639)**	**CIANI** **(*****n*** **=** **65)**	***p***
I	5% (31)	3% (2)	0.73
II	26% (164)	5% (3)	<0.01
III	19% (159)	31% (20)	0.37
IV	45% (285)	62% (40)	0.01

*CIANI, Continuous Intra-arterial Infusion of Nimodipine; WFNS, World Federation of Neurosurgical Societies Scale*.

### Adverse Events and Complications

There were 36 AEs and 49 complications during the CIANI therapy (Table 3). A total of five (14%) AEs and 13 (27%) complications were rated as mild, whereas 31 (86%) AEs and complications (63%) were rated as moderate, respectively. There were 5 (10%) severe complications. These AEs and complications, however, appeared sometimes together in one patient over the course. Thrombus formations as an example for moderate AEs at the end of the catheter were frequently detected. However, they were not always responsible for the outcome-related complications. Furthermore, pulmonary related complications were diagnosed in 12% (*n* = 8, mild). Nimodipine-associated hypotension was seen in 8% of cases (*n* = 5, mild). Additional intracerebral hemorrhage as a consequence of anticoagulation and thrombocyte-inhibiting therapy was found in 14% of cases (*n* = 9, moderate). Finally, catheter-associated cerebral infarctions as consequence of thromboembolic complications were seen in 9% (*n* = 6, moderate). Out of 65 patients treated by CIANI, 4 patients (6%) died during the treatment period and one (2%) patient died due to acute myocardial infarction (severe).

**Table 3:** Adverse events and complications in context of continuous intra-arterial nimodipine infusion.

**Severity in 65 patients**	**Adverse events** **(*****n*** **=** **patient)**		**Complications** **(*****n*** **=** **patients)**	
Mild	Catheter dislocation	6% (*n* = 4)	Pulmonary dysfunction	12% (*n* = 8)
5 AE	Catheter leak	1.5% (*n* = 1)	Systemic hypotension	8% (*n* = 5)
13 Complications				
Moderate	Catheter associated thrombus	34% (*n* = 22)	Perfusion impairment with endovascular intervention	19% (*n* = 12)
31 AE	Catheter occlusion	14% (*n* = 9)	Intracerebral hemorrhage	14% (*n* = 9)
31 complications			Thrombembolic cerebral infarction	9% (*n* = 6)
			Dissection	6% (*n* = 4)
Severe			DCI associated mortality	6% (*n* = 4)
5 complications			Myocardial infarction associated mortality	1.5% (*n* = 1)
Total 85		Total 36 (42.4%)		Total 49 (57.6%)

### Effectiveness of CIANI

The majority (*N* = 51, 79%) had no cerebral infarctions before CIANI, and 32 patients (49%) showed no cerebral infarction at the end of initial treatment or in follow-up assessment (>2 months). Furthermore, 33 (51%) displayed new cerebral infarctions in the CIANI post-treatment imaging.

### Patients Outcome and Quality of Life After CIANI

The Glasgow Outcome Scale data are summarized in Table 4. More than one third of patients in both the cohorts reached at least moderate disability and more than 20% in each cohort showed good recovery. There was no significant difference between good and moderate recovery between CIANI and non-CIANI cohorts. Severe disability was significantly more common in the CIANI group, but the vegetative state was more common in patients with non-CIANI. As for mortality, we found no significant difference between both groups (Table 4). The subgroup analysis of patients with Fisher grades III and IV showed no significant difference between the CIANI and the non-CIANI cohort. Results of the SF-36 questionnaire of patients who received CIANI treatment and its comparison to healthy population are depicted in Table 5. Patients who received CIANI have reduced QoL, if compared to the healthy population. There were only slight differences in mental health, general health, and bodily pain.

**Table 4:** Glasgow outcome scale after 6 months.

	**Conservative (*n* = 546)**	**CIANI (*n* = 55)**	***p***
Good recovery	34.0% (186)	27.3% (15)	0.39
Moderate disability	19.2% (105)	18.2% (10)	0.99
Severe disability	14.8% (81)	34.5% (19)	<0.01
Vegetative state	21.4% (117)	1.8% (1)	<0.01
Dead	10.4% (57)	18.2% (10)	0.13

**Table 5:** Results of SF-36 health-related quality of life questionnaire of patients after treatment with continuous intra-arterial nimodipine infusion.

	**CIANI, mean (SD)**	**Normal population, mean (SD)**
*n*		
Physical functioning	53.1 (35.0)	85.4 (20.7)
Role-physical	40.2 (44.8)	82.4 (32.7)
Bodily pain	69.6 (32.8)	67.4 (25.9)
General health	57.0 (23.7)	66.4 (18.2)
Vitality	42.8 (22.4)	60.0 (17.8)
Social functioning	62.1 (32.6)	86.4 (19.9)
Role-emotional	53.3 (57.1)	89.1 (26.7)
Mental health	63.8 (24.0)	72.5 (16.7)

A total of 24 patients (36%) experienced no AEs or complications during CIANI. They had an average age of 54 (SD: 11.63) years, a body mass index of 26 (SD: 6.04), predominantly aneurysms in the anterior circuit, a HH score of II (39%), and a WFNS score of 1–3 (52%) and a Fisher Score of IV (52%). The majority of patients without AEs or complications were female (*N* = 15, 65%). There is no significant difference in outcome according to the GOS between patients with and without AEs and complications (*p* = 0.691; Kruskal Wallis). Statistical group comparisons and regression analyses to identify risk factors for AEs and complications in the use of CIANI did not yield any significant results.

## Discussion

This study provides detailed information about the occurrence and frequency of AEs and complications in the context of the use of CIANI. Most AEs (86%) and complications (63%) have moderate character. Multivariate regression analysis did not identify any clear predictor related to the higher frequency of AEs and complications. Of the patients who received CIANI, 23% had a good outcome and 15% had moderate outcomes after 6 months. In comparison to QoL of patients with SAH published by our study group previously, patients who received CIANI treatment showed lower values mainly for the physical role, social functioning, and emotional role (25, 26). General health, bodily pain, and mental health were closely similar to the healthy population according to the assessment of the health-related QoL (24).

Delayed cerebral ischemia and CV are common complications in the treatment of SAH with a relevant impact on further outcomes (27, 28). CIANI became a more common salvage therapy in patients with therapy-refractory CV and DCI (8, 10, 11, 14, 29). Since this invasive treatment method is accompanied by many difficulties, our goal was to compare the treatment result after DCI and refractory CV with the frequency and severity of AEs and complications of CIANI therapy. These study results are intended to support individual decision-making and indications for CIANI therapy (risk-benefit assessment) (30).

The main aim of CIANI is to improve the clinical outcome in patients with severe refractory CV and DCI because refractory CV and DCI are often associated with severe disability or even death (31–33). Specifically, we used CIANI as salvage therapy in patients that had evidence of CV or DCI refractory to oral and intravenous therapy algorithms with nimodopine. CIANI was not used to prevent CV or DCI. Nevertheless, CIANI itself is associated with a relatively high number of side effects, AEs, and complications. We did not find any significant difference between patients who were treated with CIANI and cohorts of patients treated without intra-arterial therapy. This study is not able to confirm that CIANI is superior to other therapy options concerning outcome because the study design does not allow it. The patients were not randomized, the cohorts were not matched and the patients had different diagnostic and clinical grades of SAH, DCI, and CV. Based on that, our results cannot be generalized for all the patients with SAH (7, 12, 14). On the contrary, it is extremely difficult to perform a controlled randomized study with this question, since patients who are deteriorating because of DCI and CV have only limited treatment alternatives and withholding of a potentially curative therapy raises ethical conflicts. Careful selection of patients and defined standardized step-by-step therapy escalation are crucial in order to avoid unnecessarily aggressive therapy that might result in further complications and inferior outcomes (14, 32, 34). At this point, we suggest tight cooperation between neuroradiologists, neurointensivist, and neurosurgeon as crucial in order to schedule the CIANI to start precisely and to avoid further clinical deterioration. In addition, avoidance of sedation and ventilation might be considered in the selected cases, allowing a better monitoring and early treatment of potentially unnoticed complications and negative effects of ventilation (11).

Kieninger et al. (7) evaluated 28 patients with CIANI therapy, 60.7% of patients reached good outcomes after 6 months. Side effects such as renal replacement therapy (14% of patients) and resuscitation due to cardiac arrest (7% of patients) were evaluated in this study. Less severe were complications such as obstipation and escalation of anti-infective therapy as seen in more than 75% of patients (7). Although the study of Kieninger et al. and the study described have a different focus in the description of side effects (AEs and complications), both the studies, nevertheless, concluded that CIANI might be a helpful method to improve the outcome in patients at risk because of the refractory CV and DCI. The main difference between both studies, however, is the fact that Kieninger et al. excluded patients with CV-associated infarction from CIANI therapy, based on the higher risk of intracerebral bleeding under anticoagulation. In our study, these patients were included in CIANI treatment. This might be one explanation for the lower number of patients with good recovery. Furthermore, we had a higher number of patients with severe WFNS grades IV–V who underwent CIANI. Similarly, Bele et al. (8) showed in the analysis of 21 patients a good outcome in 76% of patients who received CIANI and significantly fewer infarctions in comparison to the control group. Only two patients suffered from thromboembolic complications in this study so there was no significant difference between the control group and the CIANI cohort (8).

Several studies confirmed the positive influence of intra-arterial nimodipine on CV and DCI (6, 8, 35). Nevertheless, it remains unclear, if possible microvasospasm and DCI might be influenced by CIANI (36). A single infusion of intra-arterial nimodipine can improve CV on imaging and functional outcome in a limited manner (6, 30, 37). This strategy is, however, related to many repetitive means of transport for DSA in patients who are mostly in the critical state (38). Furthermore, single nimodipine infusion has only a short-term durability, and therefore, its influence on DCI might be low (38).

Von der Brelie et al. (14) compared the functional outcome of patients treated by different strategies of therapy. Patients with CIANI showed poor outcomes compared with other treatments. Nevertheless, based on the selection and generally worse conditions of patients treated by CIANI, these data might have been biased. The question is, whether the patients treated by a single infusion of nimodipine have benefited from repetitive nimodipine intra-arterial administrations, or even through CIANI (37). TCD is in most cases a useful tool that is able to evaluate a good response to nimodipine treatment especially in the case of sedated and ventilated patients (37). Similar observations were published by Musahl et al. (9). In contrast to these results, Ott et al. (10) found no reliable correlation between TCD diagnosed flow velocity and CV. In the mentioned study performed by van der Brelie et al., TCD together with ptO_2_ seems to be very useful in the monitoring of CIANI therapy, which is congruent with the results presented in this study.

## Limitations

Despite this large number of patients, the generalizability of the presented results is limited because of the retrospective character of this study. Confounding cannot be ruled out. Statistical methods to deal with this fact were used. The regression analysis could not provide any additional knowledge about the results of the exploratory tests. This can be interpreted in such a way that although there are noticeable differences between patients with CIANI and non-CIANI, these do not differ or have an influence on the target variables that are of interest. A similar explanatory approach can be drawn up in the distinction between patients with and without AEs and complications with regard to risk factors for AEs and complications in the use of CIANI.

Furthermore, there is no control group, since CIANI represents a salvage therapy. The non-CIANI cohort was therefore the only relatively comparable group. Nonetheless, the groups differ in terms of their composition and also diagnostic imaging and clinical severity. There is a predominant use of CT over MRI because of the real-life character of the study. This represents a limitation in the detection of small cerebral infarctions. However, the relevance of those small infarctions that could not be displayed by CT is to discuss. Premorbid QoL data were not available. Hence, QoL data only represents results after SAH and CIANI therapy.

## Conclusion

Continuous intra-arterial infusion of nimodipine is related to a high rate of AEs and complications. These are partly due to the use of the intra-arterial catheter itself, the duration of use, the drug, and the need to use heparin and antiplatelet agents. However, these have mainly moderate character and do not directly affect the functional outcome. When patients experience CV and DCI that is refractory to standard nimodipine regimens, CIANI may be a salvage therapy. The decision-making for this salvage therapy of refractory CV and DCI should never be based on the pure presence of perfusion deficits in imaging or detection of CV in TCD. As a salvage therapy, the CIANI must be embedded in a clinical and imaging decision algorithm as an escalating procedure. A risk-benefit assessment including age or premorbid status must be included. CIANI must be seen as a step in a cascade of different other steps based on the rapid sealing of the source of bleeding to avoid rebleeding, consistent clinical and imaging monitoring at the intensive care unit, and the close monitoring of coagulation parameters during CIANI therapy to prevent bleeding complications even due to CIANI itself.

## Data Availability Statement

The raw data supporting the conclusions of this article will be made available by the authors, without undue reservation, upon request.

## Ethics Statement

The studies involving human participants were reviewed and approved by Ethikkommission der Universität Ulm. Written informed consent for participation was not required for this study in accordance with the national legislation and the institutional requirements.

## Author Contributions

AP, TK, and RK: conception, data interpretation, and manuscript writing. JS and SM: data collection and statistics. BM: statistics and interpretation. CW and BS: study conception and review. MB: data collection and interpretation. All authors contributed to the article and approved the submitted version.

## Conflict of Interest

The authors declare that the research was conducted in the absence of any commercial or financial relationships that could be construed as a potential conflict of interest.

## Publisher's Note

All claims expressed in this article are solely those of the authors and do not necessarily represent those of their affiliated organizations, or those of the publisher, the editors and the reviewers. Any product that may be evaluated in this article, or claim that may be made by its manufacturer, is not guaranteed or endorsed by the publisher.
